# Ferromagnetism and Superconductivity in CaRuO_3_/YBa_2_Cu_3_O_7-δ_ Heterostructures

**DOI:** 10.3390/ma15072345

**Published:** 2022-03-22

**Authors:** Alina Marinela Ionescu, Ion Ivan, Claudiu Locovei, Melania Onea, Adrian Crisan, Soltan Soltan, Gisela Schütz, Joachim Albrecht

**Affiliations:** 1 National Institute of Materials Physics, Atomistilor 405A, 077125 Magurele, Romania; ion.ivan@infim.ro (I.I.); claudiu.locovei@infim.ro (C.L.); melania.onea@infim.ro (M.O.); adrian.crisan@infim.ro (A.C.); 2 Faculty of Physics, University of Bucharest, Atomistilor Street 405, 077125 Magurele, Romania; 3 Department of Physics, Faculty of Science, Helwan University, Cairo 11792, Egypt; soltan@is.mpg.de; 4 Max Planck Institute for Intelligent Systems, Heisenbergstr. 3, D-70569 Stuttgart, Germany; schuetz@is.mpg.de; 5 Research Institute for Innovative Surfaces FINO, Aalen University, Beethovenstr. 1, D-73430 Aalen, Germany; joachim.albrecht@hs-aalen.de

**Keywords:** superconductor-ferromagnetic heterostructures, critical temperature, SQUID magnetometry

## Abstract

The deposition of a ferromagnetic layer can affect the properties of high-temperature superconductors underneath. We investigated the influence of ferromagnetic CaRuO_3_ on the properties of YBa_2_Cu_3_O_7-x_ (YBCO) superconducting thin films when the layers are either in direct contact or separated by a barrier layer of 5 nm SrTiO_3_. Detailed measurements of the magnetic moment of the superconductor and ferromagnet as a function of temperature and magnetic field have been performed using SQUID magnetometry. Magnetometry and relaxation measurements show that the modification of the superconducting properties of YBCO strongly depends on the interaction with the ferromagnetic layer on top. The barrier layer has a significant impact on both the supercon-ducting properties of the YBCO film and the ferromagnetic ordering of CaRuO_3_. The physical properties mentioned above were discussed in correlation with the materials’ structure determined by XRD analysis.

## 1. Introduction

Since the high-temperature superconductor YBa_2_Cu_3_O_7-x_ (YBCO) with a critical temperature *T*_c_ of more than 90 K was discovered [1], high-temperature superconductors (HTS) were taken seriously into account for the fabrication of various devices. Some of these large-scale applications where YBCO fits very well are superconducting power cables, magnetic energy-storage devices, motors, fault current limiters, and transformers [2,3,4]. Most of the superconducting properties are significantly influenced by the amount of oxygen in YBCO [5].

For an application-ready superconductor, besides a high transition temperature, high critical currents are needed; in particular, exhibiting small dissipation at high magnetic fields. To fulfil this criterion, vortices in the material have to be strongly pinned. Many possibilities to inhibit vortex motion have been found that led to an increase of the superconducting critical current density, *J*_c_ [6]. Some of the methods developed to introduce natural and artificial pinning centers are: microstructural variations [7,8], creation of oxygen vacancies [9], the addition of secondary phases [10,11], magnetic nanoparticles, or nanolayers [12,13,14,15].

Among these methods, the interaction of the superconductor (SC) with a ferromagnet (FM) leads to very interesting results [16,17,18]. If the superconductor and the ferromagnet are in direct contact, coupling phenomena such as proximity [19,20,21,22] and inverse proximity effects [23], spin-orbit [24], or dipolar coupling [25,26] can take place. For power applications, to eliminate the unwanted proximity effects [27] and increase the *J*_c_ a thin barrier layer is introduced at the SC/FM interface [28]. On the other hand, SC/FM heterostructures are also of great interest in superconducting electronics [29,30,31,32] where the properties of the ferromagnet are of utmost importance.

CaRuO_3_ (CRO) is a metal exhibiting interesting properties [33], in particular when deposited as a thin film on various substrates [34,35,36], using several deposition methods [37,38]. In respect to superconducting electronic devices, Josephson junctions have been created using YBCO and non-magnetic CRO [39,40].

Many studies have been performed to fully understand the occurrence of ferromagnetism in CRO [41,42,43,44,45,46], including studies on heterostructures with CRO [47]. Tripathi et al. [36] observed that CRO thin films deposited epitaxially on SrTiO_3_ (STO) substrates become ferromagnetic due to tensile epitaxial strain. Ferromagnetism in CRO has also been found; for example, when doping it with Ti [48]. Ivan et al. [49] managed to deposit magnetic CRO on YBCO and studied the magnetic and structural properties of the heterostructure.

In this work, we have investigated the influence of an intermediate STO layer on heterostructures composed of YBCO and CRO thin films grown by pulsed laser deposition (PLD). Using XRD measurements and detailed measurements of the magnetic moment as a function of temperature, magnetic field, and time we analyzed the influence of CRO on the superconductor and vice versa, and the role of oxygenation on their properties. We observed that CRO has good ferromagnetic properties only if it is deposited directly on YBCO and without post oxygenation.

## 2. Materials and Methods

We used pulsed-laser deposition (PLD) (Surface, Hückelhoven, Germany) to epitaxially grow thin films of CRO, YBCO, and STO. In all cases, a 200 nm thick film of optimally doped YBCO was grown on single-crystalline SrTiO_3_ (001) substrates with a lateral size of 5 mm × 5 mm. Then, for half of the samples, a 5 nm STO buffer layer was deposited on top of the YBCO surface also by PLD. The deposition of both layers was made at 830 °C in a 0.3 mbar O_2_ atmosphere with a laser energy of 67 mJ, followed by annealing in 1 bar O_2_. In the next step, 130 nm thick CaRuO_3_ was deposited at 720 °C and 0.6 mbar O_2_. The pulse laser energy was 43 mJ and annealing was performed at 0.3 bar O_2_. The heterostructures were then carefully broken into halves and a half of each was furthermore annealed at 550 °C in O_2_ atmosphere for 15 h.

**Figure 1:**
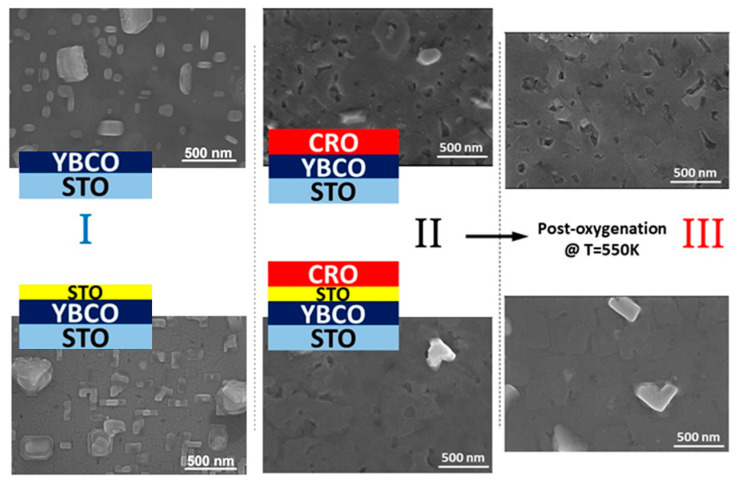
The three steps process together with the related FESEM images of the surface of each film: I: YBCO or YBCO/STO deposition on STO, II: deposition of CRO on the two samples, III: post oxygenation of the samples at *T* = 550 °C for 15 h.

A sketch showing the preparation process is depicted in Figure 1. The results in this work were subdivided into three stages of preparation: (I) reference samples YBCO (I-a) and YBCO with 5 nm STO (I-b), (II) YBCO with CaRuO_3_ in direct contact (II-a), YBCO with CaRuO_3_ separated by STO (II-b), (III) YBCO with CaRuO_3_ in direct contact oxygenated (III-a), and YBCO/STO/CRO oxygenated (III-b).

The X-ray diffraction (XRD) characterization was carried out with a Bruker D8 Advance diffractometer (Karlshruhe, Germany) operated at 40 kV and 40 mA (CuK_α_ radiation) in medium resolution parallel beam setup where reciprocal space mappings (RSMs) were recorded. Furthermore, the 2θ-ω scan was performed using a Rigaku SmartLab diffractometer (Tokyo, Japan) using monochromatized radiation Cu-K_α1_ (*λ* = 1.5406 Å). The morphology of the samples was investigated using a Gemini 500 Carl Zeiss field emission scanning electron microscope (FESEM) (Carl Zeiss Microscopy GmbH, Oberkochen, Germany), equipped with LaB_6_ filament, NanoVP mode, InLens, and SE2 detectors. The FESEM images were taken using the Inlens detector, in high vacuum.

The magnetic properties of all samples were measured with SQUID magnetometry (Quantum Design, San Diego, CA, USA) using a quantum design MPMS. In all measurements, the external magnetic field µ_0_*H* was applied perpendicular to the plane of the sample. For the measurement of the superconducting transition temperature *T*_c_, the samples were cooled in zero field (ZFC) to *T* = 5 K. Then, a magnetic field of µ_0_*H* = 1 mT was applied and the temperature was increased to 100 K while measuring the magnetic moment *m*(*T*). We extracted *T*_c_ at the temperature the diamagnetic signal vanishes. In addition, the critical current density *J*_c_ was determined from the magnetization data using the Bean relation [50] modified for a plate-like geometry [51]:

$$J_{c}=\frac{40\cdot m}{V\cdot l\left(1-\frac{l}{3L}\right)}.$$

where *m* is the magnetic moment in emu, *V*—the sample volume in cm^3^, *L* is the length and *l* is the width of YBCO thin film in cm, and the resulting *J*_c_ is in A/cm^2^.

For a more detailed analysis of vortex pinning in the superconducting film, we complemented the *J*_c_ data by measurements of the thermally activated relaxation of the magnetization. This allowed a detailed description of thermally activated depinning processes. We studied the corresponding pinning energies by long-time relaxation measurements: the sample is cooled (ZFC) to different temperatures, a magnetic field of 0.2 T is applied and the decay of the magnetic moment in time *m*(*t*) is registered for a time window *t*_w_ of 30 min.

The averaged normalized magnetization relaxation rate *S* = −Δln(|*m*|)/Δln(*t*) and implicitly the normalized vortex-creep activation energy U* = *T*/*S* can be extracted in the range where ln(|*m*|) vs. ln(*t*) is linear. This is reasonable in regions of the *T*-*H* phases diagram that are not too close to the irreversibility line (IL) and for a moderate relaxation time window, *t*_w_ averaged over *t*_w_ [52]. Typically, U* increases with temperature in the collective (elastic) vortex creep regime (where the vortex phase is ordered) [53] and decreases for plastic (dislocation mediated) creep (disordered vortex phase) [54].

Figure 1 shows a sketch of the sample preparation process, as well as the surface morphology images obtained by scanning electron microscopy (SEM). The thickness of the YBCO film is 200 nm, the CRO film is 130 nm, and the STO interlayer, where it appears, is 5 nm thick. A major difference compared to ref [49] is that the CRO was deposited after the samples were taken out of the PLD chamber and introduced again.

## 3. Results and Discussion

**Figure 2:**
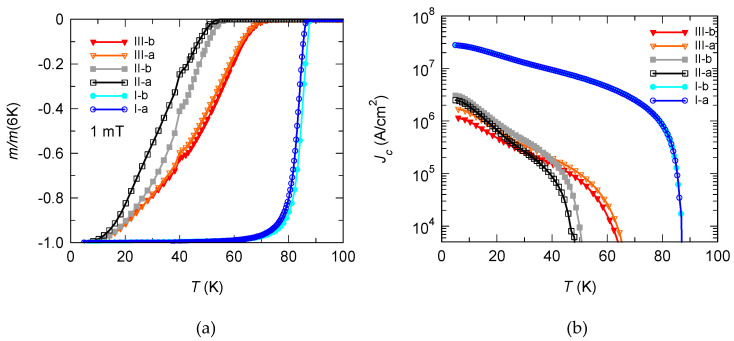
(**a**) *m*(*T*) in an applied filed of μ_0_*H* = 1 mT under ZFC conditions; (**b**) temperature-dependent critical current density *J*_c_(*T*) in the remnant state of reference samples (blue, open circles—I-a, light blue, closed circles—II-a), and the heterostructures (black, open squares—II-a; gray, closed squares- II-b, orange, open triangles III-a, and red, closed triangles III-b).

Figure 2a presents the *m*(*T*) curves for all samples in all three stages of preparation. Initially, YBCO (with and without STO), stage I, is shown in blue; after the deposition of CRO, the results are shown in black/grey (II) and, finally, after oxygenation (III), the measurements are shown in red curves. All measurements were performed in a small applied magnetic field of 1 mT. As expected, the reference samples (YBCO and YBCO/STO) have almost identical properties, exhibiting a *T*_c_ of about 86 K. After the deposition of CRO, we observe a drastic decrease of *T*_c_ for both samples to around 53 K. We managed to partially recover *T*_c_ of the superconducting film by a subsequent oxygenation at 550 °C for 15 h, after which *T*_c_ increased to 70 K.

For a better understanding of the properties of the superconductor, we measured the magnetic moment *m* below *T*_c_ to reveal the influence of the STO barrier layer and the post oxygenation on the diamagnetic response of the YBCO film. Figure 2b presents, in logarithmic scale, the temperature-dependent magnetic moment of the initial YBCO films (YBCO I-a and YBCO/STO I-b), of the samples after CRO deposition (YBCO/CRO II-a and YBCO/STO/CRO II-b) and, finally, after the post oxygenation (YBCO/CRO oxy III-a and YBCO/STO/CRO oxy III-b). The magnetic moment has been measured in the remnant state after cooling the sample in zero magnetic field to a temperature *T* = 5 K and applying an external magnetic field µ_0_*H*_ex_ = 0.3 T for several seconds. Then, the external field is reduced to zero and the magnetic moment is measured while continuously heating up until *T*_c_. The behavior of the reference samples is as expected: the high diamagnetic moment (and, implicitly, the resulting *J*_c_) decreases from low temperatures while heating up towards *T*_c_. The deposition of CRO leads to a significant suppression of *J*_c_. Interestingly, after the oxygenation, even though the *T*_c_ is partially recovered, *J*_c_ at low temperatures is further decreased. A possible reason may be the occurrence of a modification in the site of apical oxygen in YBCO [55].

In the next step, we focus on the ferromagnetism in CaRuO_3_. As shown in ref [36], CaRuO_3_ can be ferromagnetic in case of a certain thickness when grown on STO. CaRuO_3_ can be ferromagnetic as well if it is deposited on YBCO during a one-step process (first deposition of YBCO, then deposition of CRO, without taking the sample out of the PLD chamber) [49].

**Figure 3:**
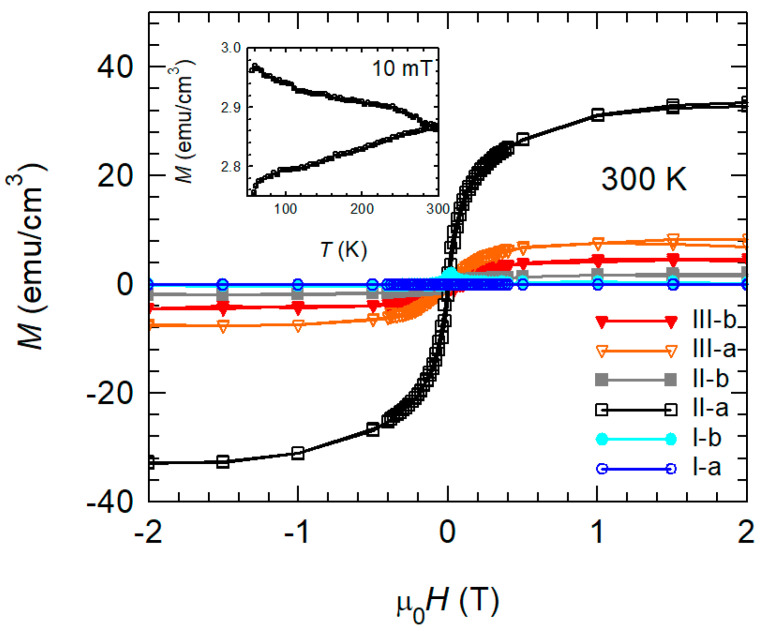
Main panel: Hysteresis loops of all the studied samples measured at 300 K. Inset: *M*(*T*) ZFC-FC above *T*_c_ in an applied field of 10 mT for sample II-a.

Our purpose was to try to preserve the good superconducting properties of YBCO while having ferromagnetic CRO on top. For the characterization of the ferromagnetic order, we measured magnetic hysteresis curves at room temperature (300 K). During analysis, the diamagnetic contribution of YBCO and STO substrate has been subtracted (Figure 3). In the case of sample II-a (CRO on YBCO), a characteristic ferromagnetic behavior is found which qualitatively corresponds to results in the literature [49]. After the reoxygenation process (stage III), the magnetic moment of CRO strongly decreases. On the other hand, if CRO is deposited on 5 nm STO on top of YBCO, the sample shows very weak ferromagnetism which is slightly increased after the reoxygenation.

To better emphasize the ferromagnetic behavior for sample YBCO/CRO, we measured the ZFC-FC temperature dependence of the magnetization in 10 mT, above *T*_c_ until 300 K. The observed bifurcation is specific for ferromagnetic materials.

As expected, if YBCO is protected by the STO layer or not, the superconducting properties of YBCO are very similar (stage I). Then, after deposition of CRO (stage II), a significant difference appears (as seen from the shape of the *m*(*T*) curves) which implies that the STO plays an important role in the YBCO. On the other hand, the only sample with good ferromagnetic properties of CRO is the one in which CRO is deposited directly on YBCO which means that STO has a big impact on the magnetic properties of CRO. The post oxygenation, although helpful for the recovery of *T*_c_ of YBCO, is damaging the ferromagnetism of CRO. Another approach should be used to keep the good superconducting properties of YBCO while maintaining ferromagnetic CRO.

Due to the complex behavior of the critical current density and for a better understanding of the role of the decoupling layer on the pinning scenario in YBCO, we performed relaxation measurements in an applied field of 0.2 T.

**Figure 4:**
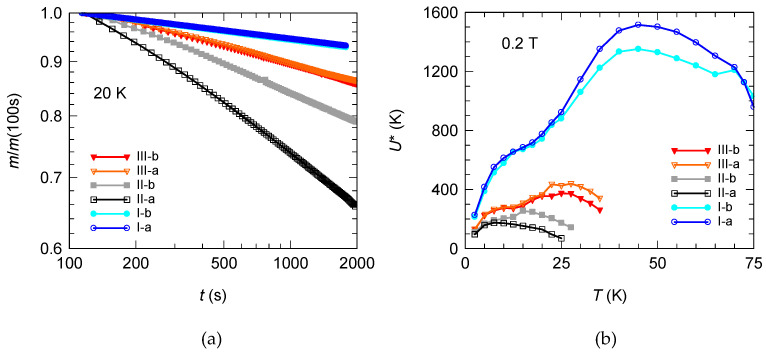
(**a**) Magnetic relaxation *m*(*t*) curves normalized to *t* = 100 s in a log–log plot for all samples at 20 K in an applied magnetic field of μ_0_*H* = 0.2 T. The normalized vortex-creep activation energy U* was extracted and represented in panel (**b**).

After each measurement, the field is reduced to 0, the sample is heated above *T*_c_, then cooled down to the next temperature. Subsequently, the field is applied and the measurement is started. Figure 4a depicts magnetic relaxation *m*(*t*) curves normalized to *t* = 100 s for all samples at 20 K which are linear in a log–log plot.

In our analysis, we excluded the early relaxation stages (*t* < 100 s) where the flux redistribution across the sample or the influence of flux flow at a very short time becomes important [56]. As it can be observed, in a log–log representation, the relaxation curves are almost linear and the normalized vortex-creep activation energy was determined using U* = *T*/*S* where *S* = −Δln(|*m*|)/Δln(*t*) (Figure 4b). It is known that the low-*T S*(*T*) maximum (in our representation the behavior of U* at temperatures under 35 K for the reference samples) is generated by the occurrence of thermo-magnetic in-stabilities (TMI) at low temperatures [56]. Then, with increasing temperature, U* increases indicating an ordered vortex phase. At higher temperatures, U* decreases, due to plastic creep. At the crossover temperature, U* can be identified with the characteristic pinning energy *U*_c_. The U*(*T*) dependence was the subject of intensive studies on YBCO films with different pinning centers [57]. However, the crossover collective creep–plastic creep always appears in DC magnetization relaxation measurements in samples with random pinning. For strongly pinned samples, the thermal energy can be neglected when *T* is significantly below *T*_c_. At low temperatures, *J* is closer to *J*_c_ because the relaxation is smaller, the effective pinning is weak and the intervortex interactions are dominant, giving rise to collective pinning. On the other hand, at high *T* the energy balance changes, because *J* relaxes faster and U* increases leading to the apparition of dislocations in the vortex system due to vortex pinning and the creep becomes plastic. Roughly, this dynamic crossover appears when the effective depth of the pinning potential well equals the vortex deformation energy [58].

As expected, the U*(*T*) dependence and the values for the two reference samples are very similar (Figure 4b). Then, after deposition of CRO, due to a decrease in critical temperature in the samples, U* also shows smaller values. Sample II-a, which exhibits ferromagnetism at room temperature due to CRO, has the smallest pinning energies. Then, after oxygenation, pinning energies increase and the two ‘bumps’ in U*(*T*) dependence are visible again for both samples. Surprisingly, in all temperature ranges, U* is higher for III-a and b samples than for II-a and b samples. At small temperatures, the differences between the heterostructures with and without STO are small and increase with temperatures.

For a deeper insight into the flux pinning properties of the superconducting film we want to distinguish between the pinning force density that is proportional to the critical current density *J*_c_ and the pinning energy U* extracted from the relaxation experiments. A more descriptive picture identifies the pinning force density with the maximum slope and the pinning energy with the depth of the pinning potential. This simplified consideration is reasonably accurate for an individual particle, namely individual vortices at low magnetic fields. This holds quite well for the results in this work.

**Figure 5:**
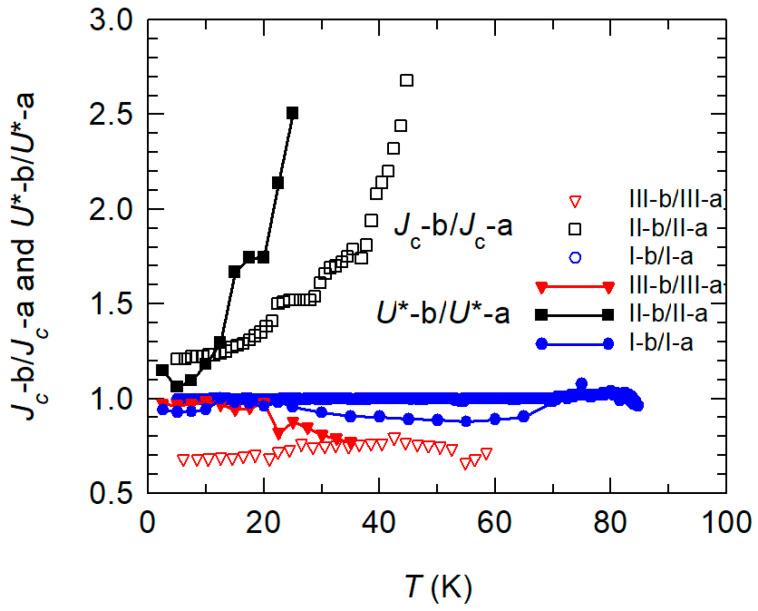
Ratio of *J*_c_ with and without STO decoupling layer for each step (*J*_c_(I-b)/*J*_c_ (I-a), *J*_c_ (II-b)/*J*_c_ (II-a) and *J*_c_ (III-b)/*J*_c_ (III-a)), open symbols, and ratio of the corresponding pinning energy U*, filled symbols.

To extract the role of the decoupling STO layer for flux pinning during three steps of preparation we consider the ratio of *J*_c_ and U* for heterostructures with and without the STO layer, respectively. Figure 5 shows the relative change of *J*_c_ and U* due to the presence of an additional decoupling STO layer for the three preparations stages I (blue), II (black), and III (red). The filled symbols refer to the pinning energy U*, the open ones describe the *J*_c_ data.

The two blue curves describing the influence of an additional STO layer on a bare YBCO film show the expected behavior. Over the whole temperature range, we find a value of about one showing that the pinning in the YBCO film is not influenced by an additional epitaxial STO film with a thickness of 5 nm. After the deposition of the CRO film, the situation changes as described by the black curves. If YBCO and CRO are electronically decoupled by 5 nm of STO, the pinning suppression is drastically reduced. Pinning remains much stronger, apparent in both *J*_c_ and U*. This result is quite obvious to explain. First, the presence of the STO layer reduces the magnetic moment in the CRO; second, there is no direct contact between the superconductor and ferromagnet. The black curves describing the effect of the separation layer exhibit values larger than 1. In addition, the observed pinning strongly increases with temperature. This is more prominent in the case of U* (filled black symbols) than in *J*_c_ (open black). This evidently shows that the description of pinning by *J*_c_ and U* is not equivalent. The more pronounced difference that occurs in U* might be explained by the suppression of superconductivity in a layer of finite thickness due to the adjacent ferromagnet. This reduces the effective length of individual vortices and, thus, the pinning energy. This does not necessarily suppress *J*_c_ in the same way, in particular when considering that also magnetic pinning provides a contribution [13].

After annealing in oxygen (stage III, red curves), the situation changes. The introduction of the STO layer leads to a decrease in pinning of about 30% leading to a ratio of about 0.7. The suppression of current transport in the YBCO film by the STO layer proves that the direct contact of both films after oxygenation has beneficial consequences for *J*_c_ and U*. The post-oxygenation has recovered the oxygen stoichiometry of YBCO close to the interface by oxygen diffusion through the STO at least partially [59]. The superconducting phase is in direct contact with the ferromagnetically ordered CRO. In this situation, magnetic vortex pinning occurs that leads to an increase of *J*_c_ and U*, respectively. A separating STO layer reduces this effect, the red curves show values smaller than 1. In the case of U*, the reduction sets in above about *T* = 25 K. This shows that thermally activated depinning of vortices is not the dominant process that limits the critical current density at low temperatures. This is consistent with the established descript of temperature-dependent critical currents in YBCO thin films and multilayers in small magnetic fields [60,61].

For a better understanding of the interaction between SC and FM, the final step of our study was the correlation of the physical properties of the heterostructures with the materials´ structure by XRD analysis.

**Figure 6:**
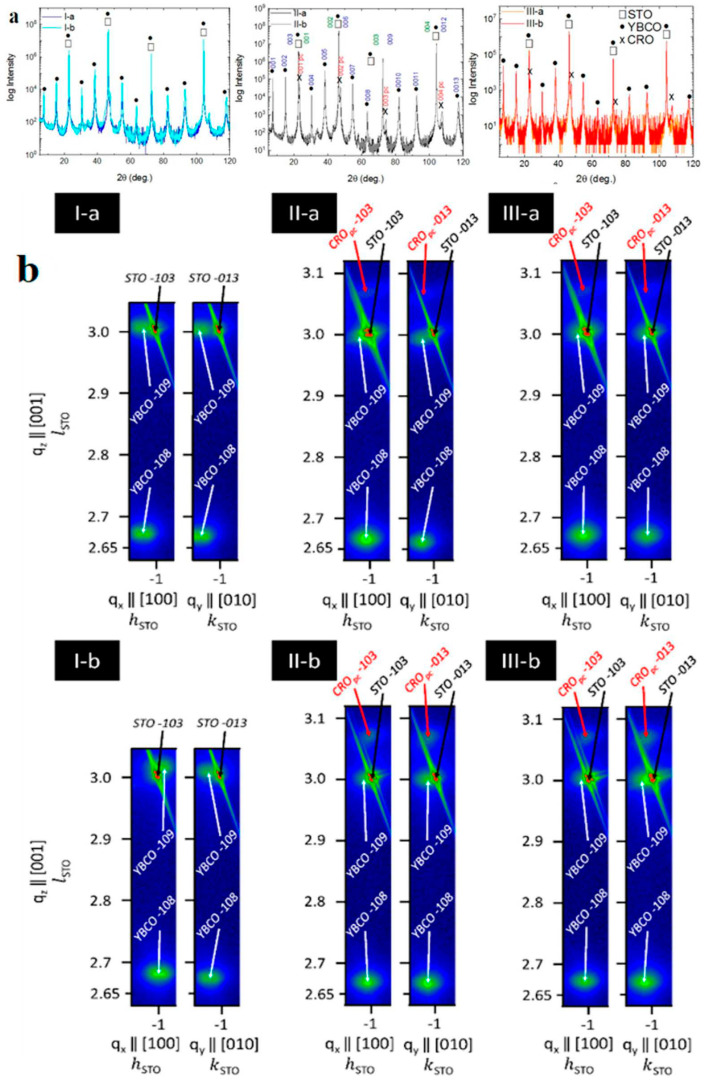
XRD characterization in: (**a**) 2θ-ω scan were the lines corresponding to multiple reflections on the (001) planes of the cubic STO substrate, of the orthorhombic YBCO structure, and of the orthorhombic CRO (in pseudocubic indexing, CROpc) are detected; (**b**) RSMs around the -103 and -013 nodes of STO substrate for all samples.

The XRD data in the 2θ-ω scan were recorded after sample alignment for miscut cor-rection of the single crystal substrate, and the results are shown in Figure 6a are the lines corresponding to multiple reflections on the (001) planes of the cubic STO substrate (ICDD # 00-035-0734), of the orthorhombic YBCO structure (ICDD # 01-086-0477), and of the orthorhombic CRO (in pseudocubic indexing, CRO_pc_) (ICDD #04-006-6459) are detected.

To show the epitaxial relationships between YBCO and CRO layers with respect to the STO substrate and to determine the in-plane structural parameters, the RSM measurements were acquired on tilted diffraction planes. The data were collected near the -103 and -013 nodes of STO to determine both *a* and *b* in-plane lattice constants of the orthorhombic YBCO. The lattice parameters calculated from RSMs are presented in Table 1. Firstly, sample I-a presents in-plane compressive strain and out-of-plane tensile strain, which is opposite to the values observed for sample I-b even though both have the highest *T*_c_ (see Figure 2b). In this reasoning, the differences observed in structural parameters of samples I-a and I-b do not have a high impact on the stoichiometry of the YBCO layer. After the deposition of the CRO layer, for all samples was observed the same value of compressive strain for the *b* lattice constant of the YBCO layer and a tensile strain in the case of *a* and *c* lattice constants. Moreover, the *T*_c_ of YBCO is smaller in these samples regardless of the presence of the STO interlayer, which may imply a modification in stoichiometry leading to an under-doped YBCO [62,63]. Differences observed in the strain of the YBCO layer and temperature-dependent magnetic moment curves from Figure 2b of samples II-a, II-b, III-a, and III-b, respectively, may be partly caused by a change in the site of apical oxygen [55]. The CRO layers show an out-of-plane compressive strain and in-plane tensile strain, respectively. Only for sample II-a, the CRO layer has an in-plane compressive strain for the *a* lattice constant, similar to the situation discussed in [49]. Thus, according to other studies [49], to achieve the ferromagnetism in the epitaxial CRO layer, an induced strain in the CRO_pc_ pseudo-cubic structure is required, but a tensile strain in both in-plane directions decreases drastically the saturation magnetization as shown in Figure 3.

However, the interaction between YBCO and CRO is complex and we took into account only some effects which appear at the interface. Probably there are also other effects such as spin diffusion, evidenced in ref [16], which will be further studied.

## 4. Conclusions

In summary, we investigated the properties of heterostructures of CaRuO_3_ (CRO) and YBa_2_Cu_3_O_7-x_ (YBCO) thin films. It has been of particular interest if the layers are in direct contact or separated by a barrier layer of SrTiO_3_. The influence of CRO on YBCO and vice versa has been studied by SQUID magnetometry. Detailed measurements of the magnetic moment of the heterostructures as a function of temperature, magnetic field, and relaxation time have been performed. It has been found that a large ferromagnetic signal is found in the case of direct deposition of CRO on YBCO, while in the case of separated layers, the magnetization is reduced. Furthermore, we observed a strong suppression of superconductivity after the direct deposition of the CRO film. A subsequent oxidation process shows that the superconducting properties are partially recovered, most likely due to an efficient oxygen diffusion through the STO barrier. The materials´ structure has been investigated by XRD analysis identifying mechanical strain to be the origin for the formation of a ferromagnetic state in CRO. The highest magnetization has been obtained when the thin layer CRO layer was deposited directly on YBCO, without further oxygenation.

## Figures and Tables

**Table 1:** Lattice constants and strain calculated from RSMs.

**YBCO-Layer**				
	**Sample**	**a (** **Å)** **Strain**	**b (** **Å)** **Strain**	**c (** **Å)** **Strain**
YBCO Bulk a = 3.817 Å b = 3.883 Å c = 11.682 Å	I-a	3.793 −0.63%	3.834 −1.26%	11.69 0.07%
	I-b	3.845 0.74%	3.905 0.57%	11.657 −0.21%
	II-a	3.834 0.45%	3.88 −0.08%	11.73 0.41%
	II-b	3.857 1.04%	3.88 −0.08%	11.705 0.2%
	III-a	3.88 1.64%	3.88 −0.08%	11.697 0.13%
	III-b	3.861 1.14%	3.88 −0.08%	11.69 0.07%
**CRO-Layer**				
	**Sample**	**a (** **Å)** **Strain**	**b (** **Å)** **Strain**	**c (** **Å)** **Strain**
CRO_pc_ Bulk pc a = 3.840 Å	II-a	3.826 −0.35%	3.883 1.13%	3.818 −0.57%
	II-b	3.861 0.53%	3.887 1.24%	3.813 −0.7%
	III-a	3.88 1.03%	3.876 0.93%	3.813 −0.7%
	III-b	3.864 0.63%	3.887 1.24%	3.812 −0.73%

## Data Availability

The relevant data for the discussion are completely supplied in the results section. Raw data is available upon request.

## References

[1] Wu M.K., Ashburn J.R., Torng C.J., Hor P.H., Meng R.L., Gao L., Huang Z.J., Wang Y.Q., Chu C.W. (1987). Superconductivity at 93 K in a new mixed-phase Yb-Ba-Cu-O compound system at ambient pressure. Phys. Rev. Lett..

[2] Foltyn S.R., Civale L., Macmanus-Driscoll J.L., Jia Q.X., Maiorov B., Wang H., Maley M. (2007). Materials science challenges for high-temperature superconducting wire. Nat. Mater..

[3] Larbalestier D., Gurevich A., Feldmann D.M., Polyanskii A. (2001). High-Tc superconducting materials for electric power applications. Nature.

[4] Seidel P., Seidel P. (2015). Applied Superconductivity: Handbook on Devices and Applications.

[5] Conder K. (2001). Oxygen diffusion in the superconductors of the YBaCuO family: Isotope exchange measurements and models. Mater. Sci. Eng. R Rep..

[6] Jha A.K., Matsumoto K. (2019). Superconductive REBCO thin films and their nanocomposites: The role of rare-earth oxides in promoting sustainable energy. Front. Phys..

[7] Yan H., Abdelhadi M.M., Jung J.A., Willemsen B.A., Kihlstrom K.E. (2005). Exponential dependence of the vortex pinning potential on current density in high-Tc superconductors. Phys. Rev. B.

[8] Jooss C., Warthmann R., Kronmüller H., Haage T., Habermeier H.U., Zegenhagen J. (1999). Vortex pinning due to strong quasiparticle scattering at antiphase boundaries in YBa_2_Cu_3_O_7-δ_. Phys. Rev. Lett..

[9] Theuss H., Kronmüller H. (1991). The influence of a point defect structure on the magnetic properties of YBa_2_Cu_3_O_7-δ_ polycrystals. Phys. C.

[10] Opherden L., Sieger M., Pahlke P., Hühne R., Schultz L., Meled A., Van Tendeloo G., Nast R., Holzapfel B., Marco B. (2016). Large pinning forces and matching effects in YBa_2_Cu_3_O_7-δ_ thin films with Ba_2_Y(Nb/Ta)O_6_ nano-precipitates. Sci. Rep..

[11] Crisan A., Dang V.S., Mikheenko P., Ionescu A.M., Ivan I., Miu L. (2017). Synergetic pinning centres in BaZrO_3_-doped YBa_2_Cu_3_O_7-x_ films induced by SrTiO_3_ nano-layers. Supercond. Sci. Technol..

[12] Rouco V., Córdoba R., De Teresa J.M., Rodríguez L.A., Navau C., Del-Valle N., Via G., Sánchez A., Monton C., Kronast F. (2017). Competition between Superconductor—Ferromagnetic stray magnetic fields in YBa_2_Cu_3_O_7-x_ films pierced with Co nano-rods. Sci. Rep..

[13] Stahl C., Walker P., Treiber S., Christiani G., Schütz G., Albrecht J. (2014). Using magnetic coupling in bilayers of superconducting YBCO and soft-magnetic CoFeB to map supercurrent flow. EPL.

[14] De Andrés Prada R., Gaina R., Biškup N., Varela M., Stahn J., Bernhard C. (2019). Controlling the strength of ferromagnetic order in YBa_2_Cu_3_O_7_/La_2/3_Ca_1/3_MnO_3_ multilayers. Phys. Rev. B.

[15] Huang S.W., Wray L.A., Jeng H.T., Tra V.T., Lee J.M., Langner M.C., Chen J.M., Roy S., Chu Y.H., Schoenlein R.W. (2015). Selective interlayer ferromagnetic coupling between the Cu spins in YBa_2_Cu_3_O_7-x_ grown on top of La_0.7_Ca_0.3_MnO_3_. Sci. Rep..

[16] Soltan S., Albrecht J., Habermeier H.U. (2004). Ferromagnetic/superconducting bilayer structure: A model system for spin diffusion length estimation. Phys. Rev. B.

[17] Samal D., Anil Kumar P.S. (2010). Evidence for decoupled two-dimensional vortex behavior of YBa_2_Cu_3_O_7-δ_ in La_0.7_Sr_0.3_MnO_3_/YBa_2_Cu_3_O_7-δ_/La_0.7_Sr_0.3_MnO_3_ trilayer. J. Appl. Phys..

[18] Sander A., Orfila G., Sanchez-Manzano D., Reyren N., Mawass M.A., Gallego F., Collin S., Bouzehouane K., Höflich K., Kronast F. (2021). Superconducting imprint of magnetic textures in ferromagnets with perpendicular magnetic anisotropy. Sci. Rep..

[19] Buzdin A.I., Ryazanov V.V. (2006). Proximity effect in superconductor-ferromagnet heterostructures. Comptes Rendus Phys..

[20] Paull O.H.C., Pan A.V., Causer G.L., Fedoseev S.A., Jones A., Liu X., Rosenfeld A., Klose F. (2018). Field dependence of the ferromagnetic/superconducting proximity effect in a YBCO/STO/LCMO multilayer. Nanoscale.

[21] Satapathy D.K., Uribe-Laverde M.A., Marozau I., Malik V.K., Das S., Wagner T., Marcelot C., Stahn J., Brück S., Rühm A. (2012). Magnetic Proximity Effect in YBa_2_Cu_3_O_7_/La_2/3_Ca_1/3_MnO_3_ and YBa_2_Cu_3_O_7_/LaMnO_3+δ_ Superlattices. Phys. Rev. Lett..

[22] Frano A., Blanco-Canosa S., Schierle E., Lu Y., Wu M., Bluschke M., Minola M., Christiani G., Habermeier H.U., Logvenov G. (2016). Long-range charge-density-wave proximity effect at cuprate/manganate interfaces. Nat. Mater..

[23] Kalcheim Y., Millo O., Di Bernardo A., Pal A., Robinson J.W.A. (2015). Inverse proximity effect at superconductor-ferromagnet interfaces: Evidence for induced triplet pairing in the superconductor. Phys. Rev. B—Condens. Matter Mater. Phys..

[24] Banerjee N., Ouassou J.A., Zhu Y., Stelmashenko N.A., Linder J., Blamire M.G. (2018). Controlling the superconducting transition by spin-orbit coupling. Phys. Rev. B.

[25] Brisbois J., Motta M., Avila J.I., Shaw G., Devillers T., Dempsey N.M., Veerapandian S.K.P., Colson P., Vanderheyden B., Vanderbemden P. (2016). Imprinting superconducting vortex footsteps in a magnetic layer. Sci. Rep..

[26] Aladyshkin A.Y., Silhanek A.V., Gillijns W., Moshchalkov V.V. (2009). Nucleation of superconductivity and vortex matter in superconductor- ferromagnet hybrids. Supercond. Sci. Technol..

[27] Chien T.Y., Kourkoutis L.F., Chakhalian J., Gray B., Kareev M., Guisinger N.P., Muller D.A., Freeland J.W. (2013). Visualizing short-range charge transfer at the interfaces between ferromagnetic and superconducting oxides. Nat. Commun..

[28] Albrecht J., Soltan S., Habermeier H.U. (2005). Magnetic pinning of flux lines in heterostructures of cuprates and manganites. Phys. Rev. B.

[29] Di Bernardo A., Komori S., Livanas G., Divitini G., Gentile P., Cuoco M., Robinson J.W.A. (2019). Nodal superconducting exchange coupling. Nat. Mater..

[30] Soltan S., Albrecht J., Goering E., Schütz G., Mustafa L., Keimer B., Habermeier H.U. (2015). Preparation of a ferromagnetic barrier in YBa_2_Cu_3_O_7-δ_ thinner than the coherence length. J. Appl. Phys..

[31] Ionescu A.M., Simmendinger J., Bihler M., Miksch C., Fischer P., Soltan S., Schütz G., Albrecht J. (2020). Soft-magnetic coatings as possible sensors for magnetic imaging of superconductors. Supercond. Sci. Technol..

[32] Alidoust M., Halterman K. (2018). Half-metallic superconducting triplet spin multivalves. Phys. Rev. B.

[33] Schultz M., Klein L., Reiner J.W., Beasley M.R. (2006). Low-temperature magnetoresistance in untwinned CaRuO_3_ films. Phys. B Condens. Matter.

[34] Tian H.Y., Wang J., Wang Y., Qi J.Q., Wong K.H., Chan H.L.W., Choy C.L. (2004). Highly c-axis oriented CaRuO_3_ thin films on LaAlO_3_ buffered Si(100) substrates by pulsed laser deposition. Phys. Status Solidi Appl. Res..

[35] Ito A., Matsumoto H., Goto T. (2007). Microstructure and Electrical Conductivity of Epitaxial CaRuO_3_ Thin Films Prepared on (001), (110) and (111) SrTiO_3_ Substrates by Laser Ablation. J. Ceram. Soc. Jpn..

[36] Tripathi S., Rana R., Kumar S., Pandey P., Singh R.S., Rana D.S. (2014). Ferromagnetic CaRuO_3_. Sci. Rep..

[37] Nair H.P., Liu Y., Ruf J.P., Schreiber N.J., Shang S.L., Baek D.J., Goodge B.H., Kourkoutis L.F., Liu Z.K., Shen K.M. (2018). Synthesis science of SrRuO_3_ and CaRuO_3_ epitaxial films with high residual resistivity ratios. APL Mater..

[38] Geiger D., Scheffler M., Dressel M., Schneider M., Gegenwart P. (2012). Broadband microwave study of SrRuO_3_ and CaRuO_3_ thin films. J. Phys. Conf. Ser..

[39] Myers K.E., Char K., Colclough M.S., Geballe T.H. (1994). Noise characteristics of YBa_2_Cu_3_O_7-δ_/CaRuO_3_/YBa_2_Cu_3_O_7-δ_ Josephson junctions. Appl. Phys. Lett..

[40] Lee S.G., Park K., Park Y.K., Park J.C. (1994). High Tc superconductor-normal-superconductor step-edge junction dc SQUIDs with CaRuO_3_ as the normal metal. Appl. Phys. Lett..

[41] Shirako Y., Satsukawa H., Kojitani H., Katsumata T., Yoshida M., Inaguma Y., Hiraki K., Takahashi T., Yamaura K., Takayama-Muromachi E. (2010). Magnetic properties of high-pressure phase of CaRuO_3_ with post-perovskite structure. J. Phys. Conf. Ser..

[42] Shirako Y., Satsukawa H., Wang X.X., Li J.J., Guo Y.F., Arai M., Yamaura K., Yoshida M., Kojitani H., Katsumata T. (2011). Integer spin-chain antiferromagnetism of the 4d oxide CaRuO_3_ with post-perovskite structure. Phys. Rev. B.

[43] Shen S., Li Z., Tian Z., Luo W., Okamoto S., Yu P. (2021). Emergent Ferromagnetism with Fermi-Liquid Behavior in Proton Intercalated CaRuO_3_. Phys. Rev. X.

[44] Bradarić I.M., Matić V.M., Savić I., Rakočević Z., Popović M., Destraz D., Keller H. (2018). Anomalous magnetic properties of CaRuO_3_ probed by AC and DC magnetic measurements and by low Ti impurity doping. Phys. Rev. B.

[45] Longo J.M., Raccah P.M., Goodenough J.B. (1968). Magnetic properties of SrRuO_3_ and CaRuO_3_. J. Appl. Phys..

[46] Chen Y.B., Zhou J., Wu F.X., Ji W.J., Zhang S.T., Chen Y.F., Zhu Y.Y. (2010). Microstructure and ferromagnetic property in CaRuO_3_ thin films with pseudoheterostructure. Appl. Phys. Lett..

[47] Chen P.F., Chen B.B., Tan X.L., Xu H.R., Xuan X.F., Guo Z., Jin F., Wu W.B. (2013). High-Tc ferromagnetic order in CaRuO_3_/La_2/3_Ca_1/3_MnO_3_ superlattices. Appl. Phys. Lett..

[48] He T., Cava R.J. (2001). Disorder-induced ferromagnetism in CaRuO_3_. Phys. Rev. B—Condens. Matter Mater. Phys..

[49] Ivan I., Pasuk I., Crisan A., Sandu V., Onea M., Leca A., Cosar C., Burdusel M. (2021). New superconductor/ferromagnet heterostructure formed by YBa_2_Cu_3_O_7-x_ and CaRuO_3_. Supercond. Sci. Technol..

[50] Bean C.P. (1962). Magnetization of hard superconductors. Phys. Rev. Lett..

[51] Gyorgy E.M., Van Dover R.B., Jackson K.A., Schneemeyer L.F., Waszczak J.V. (1989). Anisotropic critical currents in Ba_2_YCuO_7_ analyzed using an extended Bean model. Appl. Phys. Lett..

[52] Yeshurun Y., Malozemoff A.P., Shaulov A. (1996). Magnetic relaxation in high-temperature superconductors. Rev. Mod. Phys..

[53] Feigelman M.V., Geshkenbein V.B., Larkin A.I., Vinokur V.M. (1989). Theory of collective flux creep. Phys. Rev. Lett..

[54] Abulafia Y., Shaulov A., Wolfus Y., Prozorov R., Burlachkov L., Yeshurun Y., Majer D., Zeldov E., Wühl H., Geshkenbein V.B. (1996). Plastic vortex creep in YBa_2_Cu_3_O_7-x_ crystals. Phys. Rev. Lett..

[55] Ito W., Mahajan S., Yoshida Y., Morishita T., Kumagai M., Yabuta K. (1994). Influence of Crystal Strain on Superconductivity of a-Axis Oriented YBa_2_Cu_3_O_x_ Films. Jpn. J. Appl. Phys..

[56] Miu L., Mele P., Crisan A., Ionescu A., Miu D. (2014). Evolution of vortex dynamics in YBa_2_Cu_3_O_7_ films with nanorods by adding nanoparticles. Phys. C Supercond. Appl..

[57] Kuncser V., Miu L. (2014). Size Effects in Nanostructures.

[58] Blatter G., Feigel’Man M.V., Geshkenbein V.B., Larkin A.I., Vinokur V.M. (1994). Vortices in high-temperature superconductors. Rev. Mod. Phys..

[59] Ionescu A.M., Bihler M., Simmendinger J., Miksch C., Fischer P., Cristiani G., Rabinovich K.S., Schütz G., Albrecht J. (2021). Transient increase of Tc and Jc in superconducting/metallic heterostructures. Mater. Chem. Phys..

[60] Albrecht J., Djupmyr M., Brück S. (2007). Universal temperature scaling of flux line pinning in high-temperature superconducting thin films. J. Phys. Condens. Matter.

[61] Djupmyr M., Soltan S., Habermeier H.U., Albrecht J. (2009). Temperature-dependent critical currents in superconducting YBa_2_Cu_3_O_7-δ_ and ferromagnetic La_2/3_Ca_1/3_MnO_3_ hybrid structures. Phys. Rev. B—Condens. Matter Mater. Phys..

[62] Stangl A., Palau A., Deutscher G., Obradors X., Puig T. (2021). Ultra-high critical current densities of superconducting YBa_2_Cu_3_O_7-δ_ thin films in the overdoped state. Sci. Rep..

[63] Prajapat C.L., Singh S., Bhattacharya D., Ravikumar G., Basu S., Mattauch S., Zheng J.G., Aoki T., Paul A. (2018). Proximity effects across oxide-interfaces of superconductor-insulator-ferromagnet hybrid heterostructure. Sci. Rep..

